# Gestion du recouvrement des frais médicaux dans le service des urgences d’un hôpital public de la ville de Douala

**DOI:** 10.11604/pamj.2022.41.267.33053

**Published:** 2022-04-01

**Authors:** Paulin Egor Nkapnang, Norbert Marcellien Nadjie Simo

**Affiliations:** 1 Université de Douala, Douala, Cameroun

**Keywords:** Gestion, recouvrement, frais médicaux, Management, recovery, medical expenses

## Abstract

**Introduction:**

le recouvrement des frais médicaux dans les formations sanitaires est un problème qui met en mal tout le système de santé camerounais. Les hôpitaux ont de plus en plus du mal à recouvrer les frais médicaux après des prestations sur des patients. C´est la raison pour laquelle nous avons choisi de travailler dans cet article, sur la gestion du recouvrement des frais médicaux dans les services d´urgence de certains hôpitaux dans la ville de Douala; l´objectif étant d´analyser le système de recouvrement des frais médicaux en vue de l´amélioration de sa gestion efficiente.

**Méthodes:**

pour se faire, nous avons mené une étude mixte à visée analytique et transversale, l´échantillonnage était non probabiliste, avec un échantillon de 150 patients qui ont accepté de répondre librement au questionnaire et des personnels avec qui nous avons eu des entretiens sur une période de huit mois allant du 05 janvier à 30 aout 2021. Ceci dans le but de mieux comprendre le processus du recouvrement des frais médicaux au service des urgences de l´Hôpital Laquintinie de Douala.

**Résultats:**

il en ressort que plus de 60% des répondants n´ont encore souscrit à une assurance maladie pour limiter les problèmes de recouvrement et 43,33% savent ce qu´est le recouvrement de créance. La principale difficulté rencontrée dans le recouvrement est la lenteur administrative (64,67%) et le non-paiement des frais médicaux est dû à la pauvreté (48,67%) tout comme le coût élevé de ces frais (29,33%).

**Conclusion:**

même si la procédure de recouvrement suit un processus bien élaboré, les cas d´insolvabilités sont de plus en plus nombreux réduisant ainsi la capacité de les recouvrer. C´est la raison pour laquelle des réformes du système de recouvrement sont de plus en plus nécessaires.

## Abstract (en)

**Introduction:**

recovery of medical expenses in health care facilities is a problem that undermines the entire health system in Cameroon. Hospitals are finding it increasingly difficult to recover medical costs after performing patient care. The purpose of this study is to investigate the recovery system of medical expenses in the emergency departments of some hospitals in the city of Douala in order to improve its management.

**Method:**

we conducted a mixed analytical and cross-sectional study based on non-probability sampling. The study involved 150 patients who agreed to respond freely to a questionnaire and a staff with whom we had interviews during an eight-month period from January 05 to August 30, 2021. The purpose was to better understand the stages of recovery of medical expenses at the Emergency Department of the Lanquintinie Hospital in Douala.

**Results:**

more than 60% of respondents had not purchased health insurance to limit recovery problems, and 43.33% knew what debt recovery was. The main difficulty they faced in recovery was administrative slowness (64.67%) and the non-payment of medical costs due to poverty (48.67%) as well as the high cost of these expenses (29.33%).

**Conclusion:**

although the recovery procedure follows a well-developed process, there are increasing numbers of insolvencies, thus reducing the ability to recover them. That is why reforms to the recovery system are increasingly needed.

## Introduction

Les systèmes de financement de la santé affectent la disponibilité des services, qui peut s´en prévaloir, et qui peut en couvrir les coûts. Ce financement décrit plus que l´argent disponible pour la santé, il inclut aussi tous les mécanismes de la levée de fonds au paiement des services de santé. Un système de financement de la santé qui fonctionne bien s´assure que les gens peuvent avoir accès aux services de santé dont ils ont besoin sans encourir de dépenses insupportables [1] et que les ressources sont utilisées de façon équitable et efficace car les trois fonctions clefs d´un système de financement de la santé sont la mobilisation des ressources, la mise en commun des ressources et l´achat des ressources. Mais, il existe encore certaines inégalités dans la répartition des ressources consacrées aux systèmes de santé. Les pays à faible revenu comme le Cameroun ne représentent que 18% du revenu mondial et 11% des dépenses mondiales de santé [2]. Ce contexte crée une situation à laquelle, les patients ont des difficultés à pouvoir financer leur soin. De facto, beaucoup vont se trouver insolvables lorsque les paiements doivent s´effectuer après prestations médicales.

Ainsi, pour qu´un système de santé soit financé afin de limiter les risques d´insolvabilité, plusieurs stratégies ont été mis en place par les États notamment l´approche basée sur les mutuelles des santé et celle du financement basé sur les performance FBP qui ont tous été implémentées au Cameroun [3] facilitant par la même occasion l´accès aux soins et aux médicaments essentiels. En prenant le cas sur les mutuelles de santé, au Sénégal par exemple, ALENDA-DEMOUTIEZ nous apprend que seuls les salariés et les fonctionnaires ont droit à une couverture maladie institutionnalisée [4]. Sara NDIAYE va dans la même logique en faisant une analyse de l´approche mutualiste dans la gestion des maladies des indigents et montre l´importance de cette expérience pour la couverture maladie universelle au Sénégal [5]. Cet approche va permettre à chaque citoyen de participer au financement de sa santé en cotisant avant d´être malade ceci qui réduit considérablement les cas de factures impayées. Pour rentrer en possession de ces dépenses au préalable par les autorités sanitaires, un mécanisme de recouvrement doit être mis en place mais il sera confronté à de nombreux obstacles. Cet article se propose d´étudier les systèmes de recouvrement mis en œuvre dans plusieurs structures sanitaires de la ville de Douala dans l´optique de voir comment l´optimiser.

## Méthodes

La présente étude sur le système de recouvrement des frais médicaux dans certains hôpitaux de la ville de Douala était transversale qui s´est étendue sur une période de huit (08) mois allant de janvier à août 2020. Les informations étaient recueillies auprès des formations sanitaires (FOSA) et consignées sur une fiche d´enquête, puis complétées par les textes administratifs officiels organisant les formations hospitalières au Cameroun [6].

**Type d´étude:** pour se faire, nous avons mené une étude mixte à visée analytique et transversale, la méthode d´échantillonnage non probabiliste stratifiée.

**Cadre de l´étude:** l´étude s´est déroulée à l´Hôpital Laquintinie de Douala plus précisément au service des urgences. En effet, dans ce service que l´on enregistre de nombreux cas d´insolvabilité des patients après qu´ils aient reçus des soins car les patients venus en urgence doivent être pris en charge avant le paiement des frais y relatif. Ainsi, l´on y retrouve beaucoup de patients insolvables séquestrés. Un échantillon de 150 patients qui ont accepté de répondre au questionnaire et des personnels avec qui nous avons eu des entretiens; ceci dans le but de mieux comprendre le processus du recouvrement des frais médicaux au service des urgences de ces structures hospitalières.

**Conception de l´étude:** un plan stratégique de collecte des données a été élaboré afin d´identifier les cibles et mettre en place un système pour les aborder ceci dans le respect des règles d´éthique.

**Critères d´inclusion:** tout personnel, patient et visiteurs intervenant dans le système de recouvrement présent au moment de l´enquête et qui était physiquement et mentalement disposé à répondre aux questions.

**Variables étudiées:** l´âge, le sexe, la profession, la souscription à une police d´assurance santé. Des entretiens semi-directifs avec les personnels impliqués dans la chaine de recouvrement des frais médicaux et les patients en fonction de leur situation socioéconomiques et de leur médicalisation.

**Analyse et saisie:** au terme de de la collecte des données, les résultats ont été présenté sous forme de tableau et figures. Le traitement des données sera effectué à l´aide de Microsoft Excel 2013. Elles seront ensuite analysées à l´aide du logiciel EpiInfo version 7.0. Mais, seuls les résultats les plus saillants ont fait l´objet d´une analyse [7] dans le cadre de ce travail. La comparaison entre les variables qualitatives sera effectuée à l´aide du test paramétrique de Chi 2 ou de Fisher. Les différences seront considérées significativement à seuil 5% pour P < 0,05.

**Considérations éthique:** plusieurs obligations éthiques ont été respectées dans le cadre de cette étude notamment l´obtention des autorisations de collecte auprès des autorités compétentes. Ensuite, la seules les personnes consentantes ont été interrogées dans le strict respect de la confidentialité des informations reçues et de l´anonymat des répondants.

## Résultats

Cette investigation dont les résultats sont présentés en dessous a été menée aux services des urgences des hôpitaux cités plus haut sur un échantillon de plus de 180 parmi lesquelles les personnels (de sexe masculin, avec un niveau universitaire, des fonctions différentes et des expériences professionnelles variant de 18 mois à 13 ans, des domaines d´intervention divers) intervenants dans la chaine de recouvrement des hôpitaux en question et les patients (soit 48(32%) de sexe Masculin et 102(68%) de sexe Féminin) reçus dans les services concernés. Le but était d´interroger ces derniers afin de comprendre les raisons pour lesquelles ils accumulent des impayés et surtout prendre leur proposition pour améliorer le système de recouvrement dans cet établissement hospitalier. De ce fait, à la question de savoir ce qu´est un recouvrement, il ressort de la figure qui va suivre que, sur les 150 répondants, 65(43,33%) disent que c´est une activité qui consiste à obtenir le paiement d´un créancier, 43(28,67%) disent rien savoir, 27(18%) disent que c´est une activité qui consiste à payer directement les soins lorsqu´on est malade, et 15(10%) disent que c´est une activité qui consiste à se soigner, à régulariser ses frais médicaux sous pression du personnel soignant.

**Figure 1:**
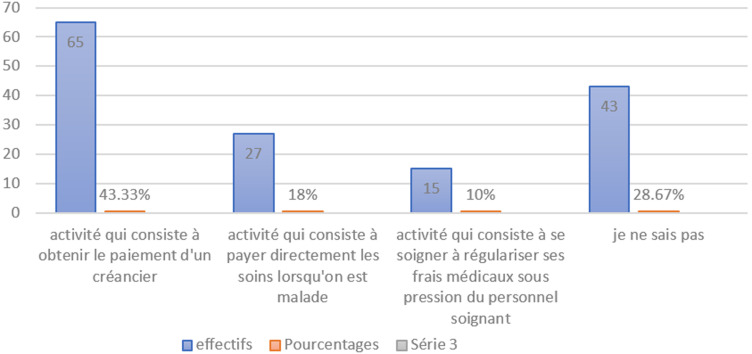
répartition des répondants en fonction de ce qu´est le recouvrement

**Figure 2:**
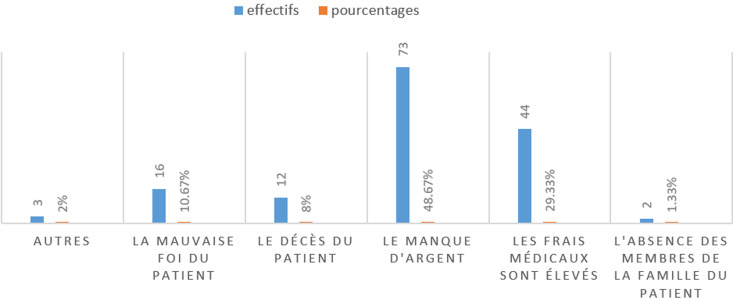
répartition des répondants en fonction de leur raison du non-paiement des frais médicaux

**Distribution de base relative aux connaissances sur les moyens de recouvrement et les facteurs favorisant les impayées:** après la collecte, l´on constate que 128(85,33%) disent recevoir un reçu informatisé, 20(13,33%) disent recevoir un reçu manuel, 2(1,33%) disent ne rien recevoir (Figure 1). Quatre-vingt dix sept (64,67%) parlent de lenteur administrative, 14 (9,33%) parlent d´absence du personnel à leur poste, 13(8,67%) parlent de problèmes techniques. Il ressort que sur les 150 répondants, 73(48,67%) parlent du manque d´argent, 44 (29,33%) disent que les frais médicaux sont élevés, 16 (10,67%) parlent de la mauvaise foi du patient, 12(3%) parlent du décès du patient, 3(2%) ont choisi autres raisons et 2 (1,33%) parlent de l´absence des membres de la famille du patient (Figure 2). En ce qui concerne l´assurance maladie comme palliatif aux problèmes d´insolvabilité, 108 répondants (72%) disent être d´accord du fait que, l´assurance maladie soit capitale au Cameroun, 28(18,67%) se disent être neutres, 14(9,33%) estiment ne pas être d´accord. Pour mieux comprendre les raisons pour lesquelles ils peuvent accumuler des impayés qui vont nécessiter un recouvrement, 75 parmi les personnes interrogées (50%) évoquent un manque de moyen financier, contre 24(17,24%) qui parlent de médicaments indisponibles à temps et 20(15,52%) parlent d´absence d´une assurance maladie tout comme seulement 6(10,35%) qui mettront en avant le manque de soutien et assistance de la famille. En parlant des politiques publiques relatives au financement de la santé au Cameroun, les répondants interrogés dans la ville de Douala notamment 105(75,84%) disent être d´accord du fait que l´absence d´une couverture est une des raisons du non payement des factures, et, 32(21,48%) qui se sont dits être neutre.

**Les différents types de recouvrement proposés aux patiens:** l´entretien réalisé avec les personnels des FOSA impliqués dans la chaine de recouvrement révèle que ces frais sont payés en espèces et parfois des virements bancaires ou chèques qui peuvent parfois avoir des remises. Le paiement en espèces a pour avantage de mieux gérer les dépenses car après l´avoir effectué, un reçu est délivré question de laisser une trace pour le paiement qui vient d´être fait. Toutefois, pour éviter de circuler avec des liquidités pouvant entrainer des agressions ou vol, le paiement par chèque peut être utilisé. Le chèque est un instrument de paiement par lequel le titulaire d´un compte bancaire donne l´ordre à sa banque de payer une certaine somme à un bénéficiaire. Un chèque n´est pas encaissable en espèces, sauf s´il s´agit pour le titulaire du compte de retirer des fonds à sa propre banque, ils sont très peu les patients qui font recours à ce mode de paiement. En plus de ces modes de paiement, le service de recouvrement accepte aussi les virements bancaires qui peuvent parfois avoir des remises par les autorités.

**Description de la procédure de recouvrement telle que appliquée dans la pratique:** la procédure standard de recouvrement est l´enregistrement de la citation, puis l´encaissement dans le système de recouvrement hospitalier. Bien plus qu´une simple réforme parmi d´autres, la tarification à l´activité est une véritable révolution du secteur hospitalier public comme c´est le cas dans la plupart des structures hospitalières étudiées dans le cadre de cet étude, inversant la logique de financement des activités cliniques et médico techniques jusqu´alors en vigueur: ce n´est plus une dotation globale annuelle mais un budget relié directement au niveau d´activité revendiqué par l´établissement qui permettra à celui-ci de financer à la fois ses dépenses courantes mais également ses investissements. Les dysfonctionnements au niveau de la chaine de facturation - recouvrement des établissements, jusqu´alors minimisés par les mécanismes de compensation budgétaire, dévoilent cruellement leur ampleur et leur impact sur l´état des finances globales de l´hôpital. En effet, si par le passé rien n´invitait les établissements sanitaires à considérer comme une priorité l´optimisation du processus de facturation, le principe même de l´action gouvernementale place dès aujourd´hui cette problématique parmi les enjeux majeurs, pour ne pas dire vitaux du secteur hospitalier, a fortiori pour les prochaines années.

Durant la ronde chaque matin, le personnel envoie les patients insolvables à la caisse pour qu´on actualise leur dette. Pour ceux qui sont déjà sortis de l´hôpital, on les relance régulièrement pour qu´ils viennent payer leur dette. Ce qui n´est pas très évident mais certains paient quand-même. En cas de décès des patients sur bon vert, le corps est déposé à la morgue et la famille doit payer avant de sortir la dépouille du défunt. Puisque qu´il s´agit d´une formation sanitaire publique, seul l´État peut poursuivre les patients, c´est-à-dire le ministère public; ceci même si un patient s´évade. Le bilan des impayés est transmis au Ministre de la Santé qui se chargera de payer mais partiellement. Dès lors, plusieurs mécanismes de recouvrement sont utilisés mais en général, ils se suivent les procédures détaillées plus haut.

**Aperçu des impayés et méthodes de recouvrement dans certaines FOSA de la ville:** dans d´autres établissements sanitaires de la ville de Douala, en dehors de ceux spécifiquement étudiés, le recouvrement des frais médicaux est presque le même c´est à dire se fait en espèce, par chèque ou même par virement bancaire. Les autres structures ici au Cameroun et dans le monde s´efforcent de recouvrer le solde intégral d´une créance des prestations hospitalières impayées ou de conclure une entente de paiement intégral à l´intérieur d´un certain délai. Lorsqu´il est impossible à ces structures de conclure une entente de paiement, elles peuvent prendre des mesures légales, qui seront impulsées par le Ministère de Santé publique. Ces mesures comprennent, mais sans s´y restreindre: la saisie-arrêt de salaires, de pensions ou de comptes à recevoir; la compensation de la créance par d´autres paiements émanant du gouvernement; la saisie des biens; l´enregistrement de la créance auprès de la Cour fédérale; l´enregistrement d´un privilège sur les biens d´un contribuable.

Ces établissements hospitaliers doivent alors poursuivre les patients insolvables (débiteurs) en justice pour recouvrer les sommes impayées. Une combinaison de procédures, aussi bien des procédures automatisées que des procédures nécessitant une intervention humaine, est utilisée pour le recouvrement des créances en souffrance. Ces procédures comprennent les lettres générées par mail, les appels téléphoniques, les visites sur place et les mesures légales telles des sommations ou injonctions à payer peuvent être mises à contribution. Le service de recouvrement de ces formations sanitaires est doté de plusieurs systèmes qui les aident à réaliser et à gérer les activités de recouvrement. Étant donné que l´entreprise recouvre les créances de tous les patients, leurs systèmes informatiques doivent être connectés aux systèmes informatiques de tous les secteurs d´activité, ce qui est en court de réalisation dans la collaboration multisectorielle entre le MINSANTE et celui des finances (MINFI).

**Les conséquences des impayées sur le fonctionnement de l´hôpital:** le non-paiement des frais médicaux est dû au manque des moyens financiers des patients et de leur famille, la baisse des activités, l´absence de sécurité sociale. Les dysfonctionnements au niveau de la chaine de facturation ou le recouvrement des établissements, jusqu´alors minimisés par les mécanismes de compensation budgétaire, dévoilent cruellement leur ampleur et leur impact sur l´état des finances globales des hôpitaux publics de Douala. Si par le passé, rien n´invitait les établissements à considérer comme une priorité l´optimisation du processus de facturation, cette problématique parmi les enjeux majeurs pour ne pas dire vitaux du secteur hospitalier, a fortiori pour les prochaines années. Ainsi, l´activité de structures hospitalières aura une baisse assez considérable vu que son fonctionnement dépend des montants collectés après chaque prestation c´est-à-dire le financement direct (…). Si un matériel est utilisé, il devra être remplacé. Les fournisseurs sont en attentes de leur paiement. Mais il est important de comprendre pourquoi des patients accumulent des impayés. Ceci a un lien étroit avec la paupérisation des populations qui est de plus en plus croissante car beaucoup n´ont pas droit à un emploi et lorsqu´ils en ont un, cet emploi n´est pas descend et régulièrement rémunéré. Les patients peuvent aussi être irresponsables.

## Discussion

**Les lieux moyens de payements utilisées:** la santé en Afrique qui a longtemps été fondée sur des systèmes de santé étatisés avec une gratuité des soins, voit aujourd´hui poindre l´émergence de systèmes mixtes avec l´implantation d´unité de soins privés liés à la paupérisation des systèmes de santé publique [8]. Cette gratuité développera certaines habitudes parmi les populations qui vont désormais avoir du mal à payer pour préserver ou restaurer leur santé d´où la récurrence des insolvabilités dans les hôpitaux du secteur public. Dans cet ordre d´idées, à propos du lieu de paiement, rappelons que 127(84,67%) disent à la caisse, 11(7,33%) disent au personnel et 128(85,33%) disent recevoir un reçu informatisé et 20(13,33%) disent recevoir un reçu manuel. Ces procédures de recouvrement avaient déjà été mises en exergue par [9], lorsqu´ils étudiaient « Le financement du secteur de la santé au Sénégal » où les mécanismes de financement participaient déjà à un recouvrement efficace. Eu égard de ces faits, notre étude s´inscrit dans un continuum des travaux précédents même si les systèmes de recouvrement ne sont pas les mêmes.

**Les facteurs associés aux impayés ici et ailleurs:** en parlant de l´aspect financier, 75 répondants (50%) parlaient du manque de moyen financier, 24(17,24%) de médicaments indisponibles à temps et 20 (15,52%) de l´absence d´une assurance maladie pour des paiements avant prestations médicales. Beaucoup évoquent le favoritisme, la richesse, le monnayage ou la possession d´un bon de PEC comme conditions pour bénéficier des soins de qualité. Le dépôt d´une caution ne semble pas peser dans la balance. Ces résultats convergent avec ceux de Joseph Parfait [10] sur le poids des dépenses de santé sur le revenu des ménages au Cameroun. Dans cette étude, OWOUNDI affirme que 51% de la population vit avec moins de deux dollars par jour, la propension moyenne de la consommation médicale totale des ménages est très élevée.

**Les effets du recouvrement des impayés sur le fonctionnement d´un hôpital:** le recouvrement des frais médicaux a une forte influence sur le fonctionnement du service et par conséquent, sur la santé des patients. Elle couvre la prévention, la formation et la recherche médicale mais également dans la prise en charge des soins des plus précaires, via la couverture maladie universelle complémentaire - CMU-C - ou l´aide médicale d´État - AME [11]. Le non-paiement des frais médicaux crée des problèmes de trésorerie et empêche le paiement des fournisseurs et du matériel ce qui ruine l´hôpital. Dans une optique néolibérale, le secteur commercial de la santé doit prodiguer des soins à des patients rentables, c´est-à-dire à ceux qui peuvent payé (indépendamment des conséquences et des inégalités sociales ainsi créées).

**Les mesures humanistes prises par les autorités sanitaires pour faciliter le recouvrement chez les patients insolvables:** les patients ayant les impayés ici deviennent des insolvables qui vont parfois s´évader, et, par conséquent, on ne peut continuer à faire les soins. Bon nombre de personnes hospitalisées dans les hôpitaux et autres structures de santé au Cameroun qui n´arrivent pas à payer les prestations médicales sont retenues dans ces hôpitaux. Dans le pire des cas, lorsque des patients décèdent avec des impayés, certains de ces corps sans vie sont confisqués jusqu´au paiement de la facture des soins prodigués. C´est pourquoi le ministre camerounais de la Santé publique, le Docteur MANAOUDA Malachie a signé en date du 14 mars 2019, une lettre proscrivant la séquestration des patients et la libération de ceux qui sont encore retenus dans les formations sanitaires fait l´objet d´une forte controverse au sein de l´opinion. Cette mesure ministérielle ne dispense pas les patients insolvables de payer leur frais médicaux, ils doivent le faire mais étant hors de l´hôpital. Ainsi, certains instruments sont utilisés pour y parvenir.

**Les difficultés pratiques rencontrées durant les périodes de recouvrements:** les instruments de recouvrement sont les factures, les bons verts, les espèces et le système de recouvrement hospitalier qui signale les impayés des patients. Lorsqu´on fait face à un patient mauvais payeur, il faut veiller à adapter la réponse à la gravité de la situation. La résolution à l´amiable est dans le bénéfice de chacun, et en tant que institution publique, cette méthode douce dans les hôpitaux publics sera toujours à votre avantage. Cette analyse est à coup sûr compatible avec celle d´Albert ZE [12] qui propose des solutions durables pour réduire voire éradiquer les insolvabilités dans les hôpitaux au Cameroun. L´enjeu du bon fonctionnement du système de santé est d´améliorer la qualité de vie des individus, des familles et des communautés. C´est pour cette raison que la performance du système de santé doit figurer en tête des priorités de l´action gouvernementale [13]. Cela a suscité au niveau de tous les EPS, un regain d´intérêt spontané pour la question du recouvrement des créances [14]. Dès lors, il est facile de se rendre compte que les impayés sont la résultante de la pauvreté dont souffre le Cameroun et de l´absence de véritable couverture sanitaire universelle.

**Limites:** le travail sur le recouvrement des frais médicaux dans les services des urgences des établissements hospitaliers de la ville de Douala peut admettre le reproche que, seuls quelques hôpitaux de la ville ont été inclus dans la recherche. De plus, les personnes interrogées se sont beaucoup laissées influencer par leur position de tantôt de gestionnaire des recouvrements, tantôt de patients en situation de précarité financière.

## Conclusion

Le recouvrement des frais médicaux dans les structures hospitalières du secteur public au Cameroun reste et demeure une véritable problématique quand on sait l´insolvabilité qui caractérise les patients ceci dû à la pauvreté qui gangrène le pays tout entier. Les hôpitaux sont donc très souvent obligés d´administrer des soins aux patients avant que ces derniers puissent payer. Mais, comme cette étude l´a montré, le recouvrement de ces fonds fait face à de nombreux problèmes car nombre de ces patients sont aux services des urgences. Étant donné qu´il existe toujours une difficulté à identifier les patients car une fois reçus dans ce service, un code leur ait attribué mais lorsque ceux-ci sortent du service, un autre code leur sera attribué sans tenir compte du précédent. Dès lors, nous avons proposé une fiche d´identification unique qui va permettre le suivi des patients dans tous les services qu´ils parcourront, question de faciliter le recouvrement des frais. De plus, il est désormais urgent d´instaurer un système d´assurance santé qui permettra aux populations de financer leur santé avant les prestations médicales dans l´optique plus se trouver obligés de recourir à ces derniers pour entrer en possession de ces frais. L´assurance maladie posera également un nouveau problème de recouvrement mais cette fois ci, il sera orienté vers les institutions d´assurance.

### Etat des connaissances sur le sujet

- *Le recouvrement amiable: cette méthode privilégie la négociation et le compromis, elle favorise le dialogue, dans le but d´emmener le débiteur à régler sa dette sans avoir à l´y contraindre; malheureusement, comme nous allons le constater, l´efficacité de l´action de cette méthode tient à l´engagement et à la bonne foi du débiteur;*
- *Le recouvrement judiciaire: au centre de cette méthode se trouvent les instances judiciaires, celles-ci sont en effet saisies, à l´effet de permettre au créancier d´entrer dans ses fonds; le débiteur sera alors contraint au règlement de sa dette [16] soit par une procédure d´injonction de payer, soit par celle de droit commun (assignation en paiement), soit par saisie immobilière;*
- *Le recouvrement par séquestration: c´est une méthode couramment utilisée dans les FOSA ici et ailleurs; elle consiste à retenir un patient insolvable à l´hôpital après sa guérison pour qu´il solde sa dette*.

### Contribution de notre étude à la connaissance

- *Elle permet l´amélioration des connaissances sur les mécanismes de recouvrement des frais médicaux au service des urgences;*
- *Elle met en évidence les causes des impayés des frais médicaux;*
- *Notre étude propose de nouvelles stratégies de recouvrement des frais médicaux et des ajustements du système de recouvrement*.

## References

[1] OMS (2000). Rapport sur la santé dans le monde 2000 Pour un système de santé plus performant, OMS, GENEVE.

[2] Lerat S, Pourtier R (1998). Atlas de la Zone Franc en Afrique subsaharienne, Monnaie, économie, société. Les Cahiers d´Outre-Mer.

[3] Silienou I Le financement basé sur la performance au Cameroun: analyse de son émergence, sa mise en oeuvre et ses effets sur la disponibilité des médicaments essentiels.

[4] Alenda-Demoutiez J (2017). Les mutuelles de santé au Sénégal face aux difficultés de coordination de leurs acteurs. RECMA.

[5] Ndiaye S (2017). Le fonds d´équité au Sénégal: analyse des mécanismes de la couverture maladie des indigents et de ses perspectives pour la couverture maladie universelle. Africa Development.

[6] Ministère de la Santé Publique (1995). Cadre conceptuel du District de Santé viable au Cameroun. Ministère de la Santé Publique. Cameroun.

[7] Benjamin Alexandre Nkoum Initiation a` la recherche : une ne´cessite´ professionnelle.

[8] Richard V (2004). Le financement de la santé en Afrique Sub-saharienne: le recouvrement des coûts. Méd Trop.

[9] Abral R, Touré B (1996). Le financement du secteur de la santé au Sénégal. Rapport phase II et rapport intérimaire.

[10] Owoundi JP (2013). The Weight of Health Expenditure in Household Income in Cameroon. Août.

[11] Kerleau M De la couverture maladie universelle aux politiques d´accès à l´assurance-maladie complémentaire: diversité des modèles et des protections.

[12] Ze Albert (2018). A17471 The importance of traditional medicine in the reduction of hypertension incidence in Subsaharan Africa. Journal of Hypertension.

[13] OMS Le Renforcement du Système de Santé. une priorité pour l´OMS.

[14] Shaw RP, Griffin CC (1995). Le financement des soins de santé en Afrique subsaharienne par la tarification des services et l´assurance. The World Bank.

[15] Cabinet FICADEX AFRIQUE (2008). Rapport définitif contrôle interne HOGGY 2007 et 2008. Cabinet FICADEX AFRIQUE.

[16] Faye D (2003). Reforme hospitalière au senegal et gestion des produits pharmaceutiques.

